# Supplementary material for: Global hypo-methylation in a proportion of glioblastoma enriched for an astrocytic signature is associated with increased invasion and altered immune landscape
Source: eLife. 2022 Nov 22;11:e77335. doi: 10.7554/eLife.77335 (PMC9681209; doi:10.7554/eLife.77335)
Supplement: Figure 2—source data 1. [file elife-77335-fig2-data1.zip › Figure_2_source_data_1/Figure_2C/homerResults/motif3.similar.html]

motif3

## Information for motif3

C
T
A
G
C
T
A
G
A
G
T
C
T
G
A
C
G
A
T
C
T
C
G
A
T
A
C
G
T
A
G
C
A
G
C
T
A
T
C
G
A
C
G
T
C
T
A
G
T
A
C
G
A
G
C
T
A
T
G
C
C
T
G
A
T
A
C
G
T
G
C
A
T
G
A
C
A
C
T
G
T
A
G
C
C
A
T
G
T
A
G
C
T
C
A
G
T
A
C
G
C
G
A
T
T
A
G
C
G
T
C
A
C
G
T
A
A
C
T
G
T
A
C
G
C
G
T
A
A
T
C
G
T
A
G
C
T
A
C
G
T
A
G
C
C
G
A
T
A
C
T
G
T
A
C
G
T
G
A
C
G
T
C
A
T
C
A
G
T
A
G
C
G
A
T
C
C
T
A
G
  
Reverse Opposite:  

A
G
T
C
C
T
A
G
A
T
C
G
A
G
T
C
C
A
G
T
A
C
T
G
A
T
G
C
T
G
A
C
G
C
T
A
A
T
C
G
A
T
G
C
A
T
C
G
A
T
G
C
G
C
A
T
A
T
G
C
T
A
G
C
G
C
A
T
C
A
G
T
A
T
C
G
G
C
T
A
A
T
G
C
A
G
T
C
A
T
C
G
G
T
A
C
A
T
C
G
T
G
A
C
A
C
T
G
A
C
G
T
A
T
G
C
A
G
C
T
T
A
C
G
T
C
G
A
A
T
G
C
G
A
T
C
T
G
C
A
T
A
G
C
T
C
G
A
A
T
C
G
A
T
G
C
A
G
C
T
C
T
A
G
A
C
T
G
T
C
A
G
G
A
T
C
G
A
T
C
  

|  |  |
| --- | --- |
| p-value: | 1e-23 |
| log p-value: | -5.363e+01 |
| Information Content per bp: | 1.456 |
| Number of Target Sequences with motif | 16.0 |
| Percentage of Target Sequences with motif | 1.36% |
| Number of Background Sequences with motif | 1.4 |
| Percentage of Background Sequences with motif | 0.03% |
| Average Position of motif in Targets | 100.6 +/- 47.0bp |
| Average Position of motif in Background | 104.8 +/- 38.7bp |
| Strand Bias (log2 ratio + to - strand density) | 2.1 |
| Multiplicity (# of sites on avg that occur together) | 1.31 |
| Motif File: | file (matrix) reverse opposite |

### Similar de novo motifs found

|  |  |  |  |  |  |  |  |
| --- | --- | --- | --- | --- | --- | --- | --- |
| Rank | Match Score | Redundant Motif | P-value | log P-value | % of Targets | % of Background | Motif file |
| 1 | 0.623 | T G A C T C G A T G A C A G C T A T G C C T G A T A C G G T A C T A C G A T C G C A G T A T C G T A G C A C G T A G T C C T A G A T G C T G C A T C A G A T C G A G T C T C G A T C A G C T A G T A C G G T C A T A G C T C G A T A G C T C A G T G A C C G T A C T A G T A C G T C G A T A G C C T G A A G T C A T G C T G A C C G T A C A T G A T G C A T G C A C T G C A G T C T A G A T G C A G C T A G T C | 1e-14 | -33.328009 | 0.94% | 0.03% | motif file (matrix) |
